# Marketing Experimental Stem Cell Therapies in the UK: Biomedical Lifestyle Products and the Promise of Regenerative Medicine in the Digital Era

**DOI:** 10.1080/09505431.2019.1656183

**Published:** 2019-09-24

**Authors:** Sonja Erikainen, Anna Couturier, Sarah Chan

**Affiliations:** a Usher Institute, College of Medicine and Veterinary Medicine, University of Edinburgh, Edinburgh, UK; b EuroStemCell, Scottish Centre for Regenerative Medicine, University of Edinburgh, Edinburgh, UK

**Keywords:** Stem cells, experimental therapies, direct-to-consumer, digital era

## Abstract

Stem cell research has attracted much public and biomedical anticipation centred on the possibility of using stem cells to treat various diseases and conditions, but the number of evidence-based therapies is currently limited. Numerous commercial direct-to-consumer (DTC) businesses are nonetheless marketing experimental stem cell therapies online for myriad medical conditions and aesthetic ailments, which has attracted critique due to safety and efficacy concerns. Existing research has largely focused on the problem of unproven therapies and regulatory pathways for addressing it. The proliferation of these experimental products must also be examined, however, in the broader socio-technological context of consumer culture and changing practices of knowledge-making in the digital era. DTC stem cell therapies have emerged as a new biomedical ‘lifestyle’ product that blurs the boundaries between ‘science,’ ‘medicine,’ and ‘consumer culture.’ In using, conceptualising and marketing stem cells, commercial businesses build on and commercially co-opt alternative epistemic and ontological frames that challenge scientific medicine. They advance promissory narratives about their potential that tap on cultural aspirations around the future of medicine and health. This is key, not only for understanding how and why these therapies have proliferated but also in conceptualising what the ‘problem’ around them actually is.

## Introduction

Translational stem cell science has attracted significant interest, controversy, and anticipation centred around the regenerative potential of stem cells and their utilisation to treat myriad diseases and ailments. Yet, apart from a small number of evidence-based therapies, evidence for the effectiveness and safety of clinical applications for stem cells is currently insufficient or in the pre-clinical stage. Despite this, recent years have witnessed the global proliferation of commercial businesses providing experimental stem cell therapies that have not undergone clinical trials but are nonetheless offered by private healthcare providers^1^ who advertise them online (Berger *et al*., 2016; Turner and Knoepfler, 2016). The emergence and success of commercial stem cell therapies is facilitated by digital technologies, which enable the creation and sharing of information and knowledge in an unprecedented scale. Healthcare businesses leverage these technologies, including major internet platforms such as Google, to market their products directly to consumers.

The emergence and abundance of stem cell businesses has attracted widespread condemnation from mainstream medical authorities who have issued warnings about the dangers of experimental stem cell therapies. Critical commentaries calling for stronger regulation and customer protection around such therapies have multiplied (e.g. Regenberg *et al*., 2009; ISSCR, 2010; McLean *et al*., 2015; Lysaght *et al*., 2017; Sipp, 2017). The success of commercial stem cell businesses suggests that large numbers of health consumers are nonetheless choosing to undergo experimental stem cell interventions marketed online, directly contravening healthcare authorities’ advice. This phenomenon takes place via an evolving digital landscape where different claims, forms of knowledge and expertise are in open competition with orthodox scientific frames of knowledge production.

The proliferation of commercial stem cell businesses has also attracted much commentary and research (e.g. Petersen and Seear, 2011; Petersen *et al*., 2015; Salter *et al*., 2017; Sipp, 2017; Murdoch *et al*., 2018). This has included efforts to document the number and distribution of the businesses and types of treatments marketed in the USA (Turner and Knoepfler, 2016) and across the globe (Berger *et al*., 2016). Existing research has primarily focused on documenting and analysing the scope of the problem, where the ‘problem’ is usually taken to be inadequate safety, efficacy, and lax regulation of experimental therapies. The key concern is potential and actual exploitation of vulnerable patients who choose to undergo and pay for therapies even though the therapies’ effectiveness and safety is not evidenced, and the knock-on effects on aftercare this may have for healthcare infrastructures.

A closer examination of the websites where stem cell therapies are advertised, however, highlights that the broader discursive and epistemic frameworks that are mobilised by businesses marketing experimental stem cell interventions are important in making sense of this growing market. These frameworks, which concern the nature of knowledge and how it is produced, also relate to the socio-medical and techno-scientific context of digital media, which is key for understanding how the businesses operate. A clue towards this end emerged from Berger *et al*. (2016) and Turner and Knoepfler’s (2016) research including on the types of stem cell therapies marketed by commercial businesses. Their research indicates that a large proportion of the stem cell therapy market is focused on ‘cosmetic’ or ‘aesthetic’ therapies, rather than on what have been defined as ‘medical’ treatments. While this does not distract from the importance of analysing the safety and efficacy of experimental medical therapies, it is interesting that this characteristic of the market has attracted little exploration and scrutiny.^2^

We offer a case-study of the UK-based commercial direct-to-consumer (DTC) online market of experimental stem cell therapies, in order to explore the following questions: how are ‘stem cells’ conceptualised and represented to consumers? What epistemic frames guide these conceptualisations and representations, and how do they fit within the current landscape of science, medicine, and consumer culture?

Our aims are twofold: firstly, while we map the scope of the UK market, our objective is less to document the number and distribution of the businesses and more to use this mapping as background for examining how the digitally mediated market itself offers an emerging alternative discursive and epistemic sphere to conventional evidence-based medicine. Whereas existing research has primarily taken the problem around commercial stem cell therapies to be insufficient regulation, safety, efficacy, and consumer exploitation, we aim to look beyond this conceptualisation of the problem. We aim to shed new light on the techno-scientific and medico-cultural context within which the therapies have been commercialised. This is because the currently dominant framings of the problem can have the effect of constraining the scope of analysis in ways that limit our ability to properly account for the context that foregrounds how these therapies are marketed.

Secondly, we aim to contribute to existing STS literature on the nature and implications of knowledge-making in the digital era. We do this by exploring how digital environments mediate the co-existence of plural knowledges and the emergence as well as commercial co-optation of alternative epistemic frameworks that challenge scientific medicine. The epistemic frames used by commercial stem cell therapy businesses exemplify a blurring of the boundaries between medicine and consumer culture. Experimental DTC stem cell therapies can be understood as a new kind of biomedical ‘lifestyle’ product that represents the fragmentation of conventional scientific authority. This perspective also broadens the scope of current discussions and debate around DTC stem cell therapies, as it offers a contextualising angle that should be accounted for in developing responses to the proliferation of experimental stem cell therapies.

## Analytical Perspectives: Medical Knowledge (Making) in the Digital Era

Digital technologies are changing how patients and consumers use, access and interpret medical information, and how they communicate about and purchase healthcare services and products (Lupton, 2013; Sosnowy, 2014; Saukko, 2018). Digital media have exponentially expanded individuals’ access to both conventional and alternative information about medicine and health. The effect is that medical knowledge (including the production and critique of this knowledge) can no longer be seen as the exclusive purview of trained medical and healthcare professionals. The plural and easily accessible information sources and knowledge claims that co-exist in online environments have made medical information open to re-interpretation and re-framing in unprecedented ways. Individuals can (re)search and evaluate this information for themselves while commercial healthcare service providers have access to new ways of marketing their products via DTC advertising, which can bypass mainstream medical information channels. This includes the unprecedented capacity of commercial businesses to distribute information about emerging but not (yet) clinically proven therapies, including experimental stem cell interventions.

Concurrently and relatedly, it has been argued that the ‘digital era’ in which we increasingly live is characterised by growing scepticism towards conventional scientific medicine. Top-down forms of medical knowledge production and hierarchical or clinician-centred models of the clinical encounter (where medical professionals are accredited with epistemic superiority) are becoming increasingly contested (Lupton, 2012). This is reflected in the drive towards patient-centred healthcare and emergence of phenomena like ‘citizen science’ (see Prainsack, 2014). These suggest that actors outside the academy and the medical profession, including patients and consumers, occupy increasingly active roles in the production and interpretation of scientific and health knowledge. They can also construct alternative understandings of their own health (Neff and Nafus, 2016; Saukko, 2018).

The emergence of DTC medicine (including stem cell therapies) is a related phenomenon. It mirrors the fragmentation of top-down forms of medical knowledge-making and pluralistic modes of health and knowledge in the digital era, but it also represents the commercialisation of knowledge pluralism. The online search technologies (see Mager, 2012) that consumers primarily use to find and access DTC services are inscribed with capitalist power dynamics: search engines including Google use tactics like user profiling to catalogue information about the interests and desires of individuals and groups based on their online histories and behaviours. This can in turn be commodified by first selling the profiles to commercial and advertising businesses and then marketing them back to consumers via user-targeted advertising (Mager, 2012). Relatedly, the parameters of the digital environments in which DTC medicine operates facilitate the commodification of patients’ and consumers’ interests and hopes (Saukko, 2018) including around experimental stem cell interventions (Petersen and Seear, 2011).

As others have analysed, health consumers choosing to undergo experimental DTC stem cell therapies often articulate their decision in terms of the hope that these interventions are perceived to carry for living a healthier or better life (e.g. Rachul, 2011; Petersen *et al*., 2013, 2015). DTC stem cell businesses in turn harness this hope in the strategies they use to market their products (Petersen and Seear, 2011). This is connected with the future-oriented nature of the stem cell therapy market: experimental stem cell interventions, like many other new and emerging biotechnologies (see e.g. Brown *et al*., 2000; Moreira and Palladino, 2005; Broer and Pickersgill, 2015), are characterised by expectations about a better, healthier future. This future is seen to be enabled by a new wave of medicine – expectations that are attached to new biotechnologies are their future promise. These ‘promissory narratives’ (see e.g. Brown *et al*., 2000) tend to utilise a rhetoric of hope whereby scientific research and experimentation are justified by the promise of new treatments or miraculous medical innovations (Moreira and Palladino, 2005).

The discourses and metaphors that DTC stem cell businesses mobilise to market their products not only enact promissory narratives but also capitalise on these narratives. They position health consumers as agents who can contribute to realising better therapeutic futures by investing in experimental (stem cell) medicine today. Indeed, much social analysis of DTC medicine in its many manifestations (e.g. Saukko *et al*., 2010; Dumit, 2012; Prainsack, 2014) points towards a tension between patient and consumer empowerment rhetoric promoted by DTC businesses and the businesses’ commercial interests.

Notably, Saukko (2018) has argued that commercially promoting medical knowledge as open, not only to consumers’ contributions but also to (re-)interpretation by consumers themselves configures this knowledge as ‘tentative.’ This is intertwined with the promotion of commercial digital healthcare as more participatory or empowering than traditional healthcare models. Instead of being framed as fixed facts that are disseminated by scientific experts who know better, medical information is re-configured as ‘in-formation’ in ways that enable dynamism in how it is understood and mobilised by individuals. This is related to the changing status of facts and expertise in contemporary digital societies: it has been argued that it can no longer be presumed that publics’ respect for facts can be secured via the authority of experts legitimated by agencies outside the public domain (Marres, 2018).

Contextualised by the changing dynamics of knowledge (making) in the digital era, Saukko *et al*. (2010) have also argued that we are witnessing the emergence of a new, digitally mediated social marketing space for biomedical ‘lifestyle’ products which blur the space between medicine and consumer culture. The emergence of these ambiguously situated products vis-a-vis medical and consumer goods is part of broader phenomena through which the conventional boundaries of science and medicine are being re-drawn (Nowotny *et al*., 2001). Indeed, Saukko *et al*. (2010) argue the emergence of these ‘hybrid’ medico-cultural entities and the transformation of the boundaries of what does or is taken to constitute science and medicine is symptomatic of our current historical times.

In what follows, we will build on and contribute to existing analyses of knowledge-making, commercialised (re)interpretation and (promissory) uses of science in the digital era. We do this by focusing on stem cell businesses and how they configure (the meaning and value of) their products through their marketing strategies and portrayals. In particular, we build on Saukko *et al*. (2010) to argue that commercial experimental stem cell therapies exemplify the blurring of science, medicine, and consumer culture in ways that have implications beyond the safety and efficacy problem of commercial stem cell interventions.

## The Landscape: Stem Cell-based Therapies in the UK and Beyond

While there has been significant research investment in clinical applications for stem cells – indeed, the UK Clinical Trials Gateway (UKCTG, 2018) contained 191 recruiting stem cell therapy trials at the time of writing – the vast majority of proposed therapies are not supported by existing scientific evidence (Sipp, 2017). In the UK, the National Health Service (NHS) largely sets the standards for accepted medical treatments. It only offers haematopoietic stem cell transplants (derived from bone marrow or blood) as part of some cancer and blood disorder treatments (e.g. leukaemia, lymphoma, and sickle cell anaemia). Recently, some NHS hospitals have also begun to offer haematopoietic stem cell transplants for some conditions. These include relapsing-remitting Multiple Sclerosis failing alternate approved therapy, albeit with strict treatment access inclusion and exclusion criteria (NHS, 2017). These new uses apply well-established treatment protocols in bone marrow and blood transplantation rather than new treatment protocols.

Commercial businesses, however, offer stem cell therapies for a variety of diseases and afflictions that are not offered (nor approved) by public and mainstream healthcare providers and authorities. These therapies have often not undergone clinical trials nor are they derived from the well-established treatments, but they are nevertheless accessible and marketed directly to customers via digital platforms. Digital media provide commercial healthcare providers with a marketing avenue and customer base that seems willing and able to pay for and even travel beyond borders to receive experimental stem cell therapies. Indeed, ‘stem cell tourists’ have received much commentary, especially from the perspectives of medical tourism and healthcare commercialisation in the global context (e.g. Song, 2010; Petersen *et al*., 2013; Petersen *et al*., 2017).

Due to scarce evidence for most stem cell therapies’ effectiveness and safety (and, in some cases, implausible scientific basis), mainstream healthcare authorities working within evidence-based epistemologies have widely condemned these therapies and the businesses providing them. The consequence has been that boundaries have been drawn between ‘legitimate’ and ‘illegitimate’ or ‘proven’ and ‘unproven’ therapies, where the boundary has primarily focused on the existence and lack thereof of evidence from clinical trials (Petersen *et al*., 2015; Sleeboom-Faulkner, 2016). Indeed, the NHS in collaboration with other healthcare authorities including the UK Department of Health have issued warnings about ‘rogue clinics’ offering ‘risky stem cell treatments’:

stem cell clinics around the world are exploiting patients … Despite the experimental state of many stem cell therapies, they are being sold over the internet directly to patients. … There is concern these companies are putting patients with often very serious and terminal conditions at further risk with untested treatments, while extracting substantial payment from them. (NHS, 2008).

The International Society for Stem Cell Research (ISSCR) has also issued guidelines for prospective stem cell patients, instructing them to evaluate stem cell therapy providers against conventional evidence-based scientific criteria (SCR, 2008). The ISSCR additionally published a report condemning unproven stem cell treatments, urging for their stronger national and international regulation (ISSCR, 2010).

Until recently, discussions around these therapies focused on lenient regulatory systems in middle-income countries including China and India. These were taken to be fuelling a movement of stem cell tourists from Western countries to emerging economies in search of alternative therapies not available in more restrictive high-income jurisdictions (Kiatpongsan and Sipp, 2009). A key regulatory issue for the global stem cell therapy market is that much of it operates outside orthodox (i.e. evidence-based, marketing approvals-compliant) research and innovation governance (Salter *et al*., 2017). Mainstream scientific influencers have argued that uninformed patients and health consumers may be exploited if appropriate regulatory oversight is not imposed and implemented for so-called unproven stem cell interventions. Stronger regulation and oversight, they argue, are needed to protect patients’ and consumers’ health, mitigate risks, and safeguard informed choice (Regenberg *et al*., 2009; ISSCR, 2010; McLean *et al*., 2015; Lysaght *et al*., 2017).

Yet, experimental stem cell therapies have become increasingly commercially available also in high-income countries including the UK, which indicates that their global proliferation cannot be reduced to weak regulation (alone) (Lysaght *et al*., 2015). Berger *et al*. (2016) have shown that the marketing of stem cell-based therapies is now skewed towards businesses in high-income economies with relatively strong regulatory systems. The largest distribution located in the USA. Indeed, Turner and Knoepfler (2016) identified 351 US-based businesses. Salter *et al*. (2017) have argued that emphasis on enforcing regulation over predatory clinics, combined with the presumption of a uniformed customer-base, fails to appropriately account for the complexities of the market.

Mainstream medical and scientific authorities often take for granted the clinical trial process that is required to establish the safety and efficacy of clinical applications. This process is, however, time consuming and frustrates demand for the speedy development of, and access to, therapies for conditions for which no evidence-based interventions currently exist (Salter *et al*., 2017). Commercial healthcare providers fill the gap between demand and therapy options, including by moving from basic research directly to clinical application.

Relatedly, others have shown how consumers themselves see their choices as informed decisions to undergo experimental interventions rather than perceiving themselves to be unfirmed. These choices are reached by weighing risks against possible benefits and hope that the interventions carry, which suggests that health consumers’ decision-making might be governed by different priorities and logics than those assumed by mainstream evidence-based medicine (Rachul, 2011; Chen and Gottweis, 2013; Mazanderani *et al*., 2017).

As Sleeboom-Faulkner (2015, p. 1) has also suggested, then, there is a need to ‘look beyond regulatory exteriors’ and towards the ‘cultural and socio-political context of debates on regulation’ of experimental stem cell therapies. This is so especially because the digitally mediated discursive space around them is characterised by competing agendas and truth claims about stem cells’ value and potential. These characteristics are not restricted to the discursive space around stem cell therapies but exemplify broader trends around the intersection of new biotechnologies and digital media: debates around anticipatory governance of commercial genomics, for example, have also assumed polarised tendencies where consumer protectionism is pitted against consumer empowerment (Prainsack *et al*., 2008). The experimental stem cell therapy market and related disputes highlight how orthodox forms of medical and healthcare provision and governance can be and are challenged by consumers’ and service providers’ agency enacted online.

## Methodology

To our knowledge, the only existing documentation of commercial UK-based stem cell therapy providers was produced by Berger *et al*. (2016) as part of their study on the global distribution of DTC stem cell therapy businesses. They identified 12 websites of UK-based businesses, but do not provide URLs. While our primary focus was qualitative thematic analysis of UK businesses’ websites, we thus first needed to identify the businesses.

We based our approach on Turner and Knoepfler’s (2016) study on DTC online marketing of stem cell therapies in the USA. We conducted a total of 50 Google search engine key word searches, using terms including ‘stem cell treatment,’ ‘stem cell therapy,’ and ‘stem cell clinic’ usually combined with the regional identifier ‘UK.’ We also searched for stem cell types (e.g. ‘adipose’), specific medical conditions (e.g. ‘multiple sclerosis’) and fields of specialisation (e.g. ‘orthopaedic’). Many businesses were identified by examining advertisements that accompanied search terms. For each term, we initially reviewed 10–15 pages of search results unless less than 10 pages were retrieved. Later, we reviewed between 7–10 pages as new searches decreasingly retrieved new businesses. We completed data collection when our searches no longer retrieved new businesses.

The internet searches were conducted in February and March 2018, making our study a snapshot of UK businesses operational at that time. We aimed to identify commercial UK businesses advertising experimental stem cell therapies directly to consumers in exchange for payment. We consequently excluded not-for-profit clinical trials^3^ and NHS-affiliated clinics offering stem cell therapies according to NHS procedures, because we took the NHS as key UK authority in delineating the scope of legitimate(d) therapies. However, we included private businesses advertising the same stem cell therapies as the NHS, but with more lenient eligibility criteria. Regulatory compliance was not an inclusion/exclusion criterion, and our findings cannot be used to assess this factor, nor do we make empirical claims about the efficacy or safety of the experimental therapies.

Some businesses based outside the UK had offices but not treatment facilities in the UK, and we only included businesses advertising stem cell interventions delivered in the UK. We also excluded businesses advertising (only) mail-order stem cell products (e.g. cosmetics); research organisations and companies not offering therapies directly; veterinary businesses; and stem cell biobanks (e.g. cord blood and tooth).

Websites of businesses meeting our inclusion criteria were downloaded using the SiteSucker application. Information including the business’ name, website URL and preliminary website content (e.g. kinds of therapies offered) were entered into a dataset. We also documented the location and number of individual facilities operated, as many businesses had a single website but multiple locations across the UK administering stem cell therapies. We produced two maps to visualise this data using the Google MyMaps tool, assigning each individual facility with a pin.

We then conducted qualitative thematic analysis (see Braun and Clarke, 2006) of all websites, aiming to identify and examine patters in the semantic content of the website and the underlying ideas and discourses that foreground and shape the semantic content. This analysis was used to derive key themes, which we discuss in this paper. In most cases we only analysed the subpages our search terms retrieved and the home and ‘about us’ pages to foster a manageable dataset, except when initial analysis indicated that stem cell- and related therapies where the business’ core focus. In these cases, we analysed the whole website.^4^

Existing studies undertaking similar mapping efforts and quantitative website content analyses of commercial stem cell businesses (Berger *et al*., 2016; Turner and Knoepfler, 2016) have used coding approaches with pre-defined categories (e.g. of stem cell types or medical specialities, like ‘adipose’ and ‘orthopaedic’). We did not follow this approach, because we aimed to analyse the language used and underlying contexts in which the notion of stem cells was mobilised. This is why we applied qualitative thematic analysis enabling an in-depth, ‘rich’ account of the data without presuming pre-existing conceptual or theoretical commitments (Braun and Clarke, 2006). Indeed, UK businesses mobilised multiple and inconsistent terms, ideas and values to describe the purported stem cell therapies offered.

Following Turner and Knoepfler (2016), we aimed to mitigate the Google page ranking bias by reviewing up to 15 results pages, but it is probable that we missed some businesses using marketing phrases different from the key words we used. By restricting the analysis on the subpages, home and ‘about us’ pages for most websites, it is also possible that we missed relevant information provided elsewhere on the websites.

## Mapping the UK Stem Cell Therapy Market

**Figure 1.**
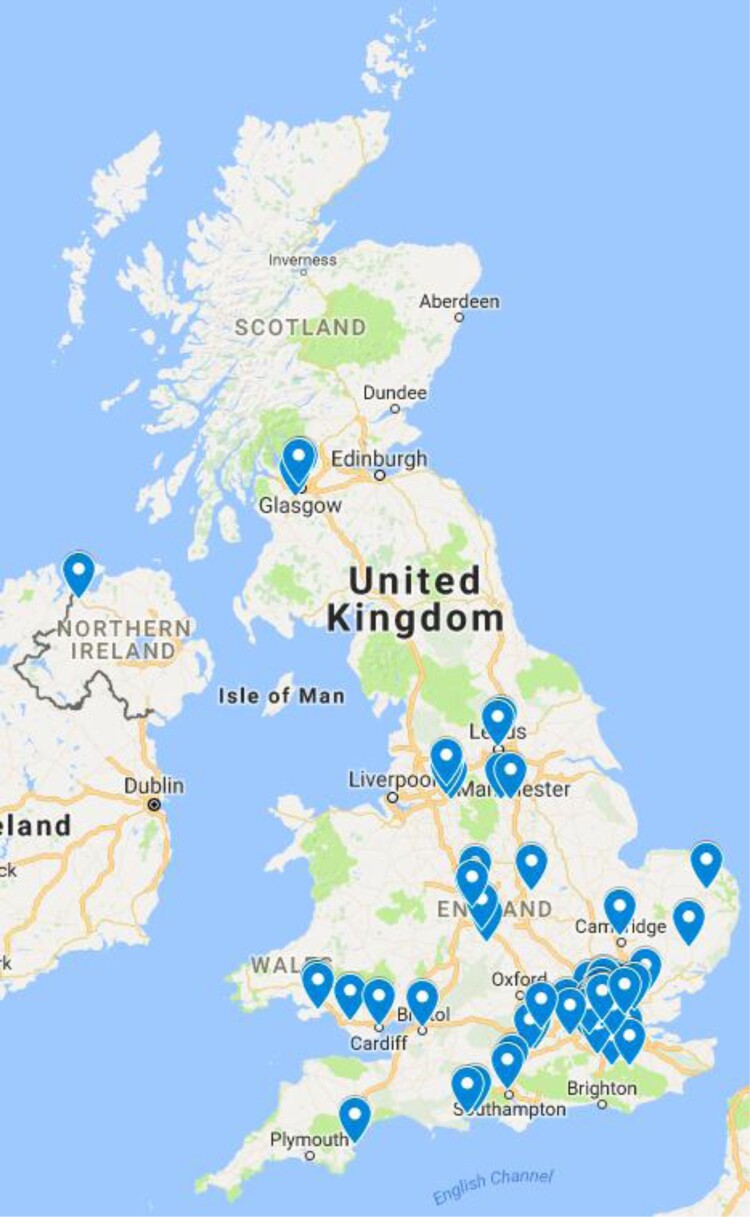
Map of UK stem cell clinics.

**Figure 2.**
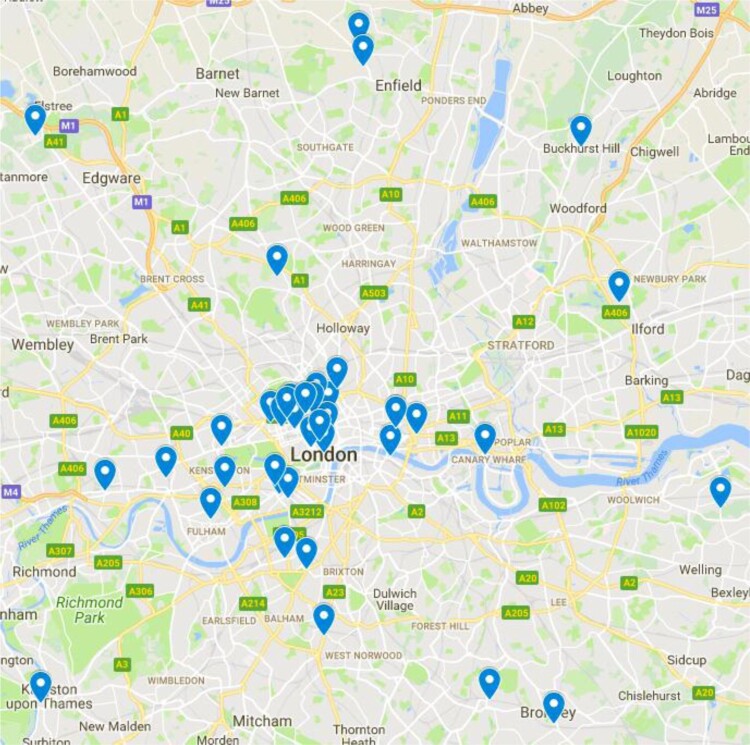
Map of London stem cell clinics.

We identified 71 websites of businesses marketing purported stem cell therapies online directly to consumers and 106 individual facilities across the UK where the therapies are administered. Notably, these numbers significantly differ from the 12 UK businesses identified by Berger *et al*. (2016) perhaps partly due to different search strategies and inclusion criteria. The facilities were concentrated in larger cities, with London hosting by far the highest number of facilities. 28 were clustered in the London West End areas of Marylebone, Covent Garden, and Fitzrovia which have an association with wealth and private healthcare (Rappaport, 2000). 15 facilities were located on Harley Street alone, which is well-known as an epicentre of elite healthcare and medicine that also has a history of attracting criticism focused around concerns over questionable scientific integrity and ‘bogus doctors’ (Humphrey, 2004). Indeed, it has been suggested that Harley Street’s association with alternative medicine and dubious practices provides an ideological focus of distaste for orthodox medical practitioners: Harley Street constitutes a useful ‘other’ for orthodox medicine to unite against (Humphrey, 2004) (Figures 1 and 2).

Most businesses across the UK use the stem cell therapy label in marketing therapies based on what are called adipose or fat stem cells (20 businesses). Therapies based on cells from bone marrow (13) and blood (8) were also common, some advertising both. Plant stem cells (4), ‘placenta’ and ‘umbilical cord’ tissue-derived stem cells ‘harvested from sheep’ (2) and ‘autologous corneal limbal stem cells’ (1) were also marketed. Many businesses (25) did not specify the biological origins of stem cells, but made generic claims about stem cell therapy or treatment, or therapies claimed to promote or rely on stem cells. Several businesses (12) marketed stem cell therapies using proprietary technologies with labels such as ‘Lipogems’ or ‘FAMI,’ where the technologies were key to the marketing strategy. Emphasis on trademarked technologies was a notable characteristic overall in how the therapies were framed for consumers.

Corresponding with Turner and Knoepfler’s (2016) findings in the USA, many UK businesses advertise Platelet Rich Plasma (PRP) therapies as a form of stem cell or stem cell-based therapy. PRP is a portion of the plasma fraction in autologous blood that has a high platelet concentration (Marx, 2001) and PRP therapy generally involves injecting this into an area of the body to promote tissue repair. Many businesses market PRP therapy by making claims to the ability of PRP injections to incite or promote stem cell action. While Turner and Knoepfler’s study excludes businesses only marketing PRP therapies, we included them if PRP was framed as stem cell therapy, or if they made claims to stem cells as part of the marketing strategy (17 businesses used this approach).

While stem cell therapies were marketed for myriad conditions and ailments, aesthetic and what can be classed as orthopaedic interventions involving musculoskeletal issues were most common. Many advertised therapies for both musculoskeletal and aesthetic ailments or other combinations of speciality and procedures in ways that muddied conventional distinctions between cosmetic and medical interventions. Most commonly advertised were interventions usually defined as cosmetic (often marketed in combination with other therapies) (52). Musculoskeletal conditions and related pain management were also common (15). Other therapies included so-called sexual dysfunction, eye conditions, and cancers.

While the number of UK businesses we identified significantly differs from Berger *et al*. (2016) findings, much of our data corresponds to their study on the global market. They also found that stem cell interventions were most commonly marketed for aesthetic factors, with several websites advertising stem cell therapies for multiple seemingly unrelated conditions. Stem cell therapies for conditions beyond the strictly medical (i.e. aesthetic, cosmetic) evidently occupy a significant portion of the market in the UK and beyond.

## Regeneration and Rejuvenation

The overarching interpretive framework that UK businesses mobilise in marketing experimental stem cell therapies harnesses and commercially re-frames biomedical models of ‘regenerative medicine.’ Stem cell therapies are primarily marketed as a form of ‘regenerative’ or ‘rejuvenation’ therapy, whereby the regeneration and rejuvenation concepts are used to make comprehensible as well as to advertise stem cells’ potential. The Centre for Advanced Facial Cosmetic and Plastic Surgeries (2018) represents this potential as follows:

the principle [is] to use your own body’s natural reparative and healing mechanisms … In the future [treatments] may be driven by triggering natural reparative mechanisms in the body that are able to regenerate and repair themselves, this is the basis of Stem-Cell based therapy and Regenerative Medicine.

The SW11 clinic (2018) states that ‘Stem cells have the ability to self-renew or multiply, meaning that they can repair and replace tissue in the human body. In other words, stem cells have the power to heal.’

The regeneration and rejuvenation concepts in this context derive from and are intertwined with broader discourses around regenerative medicine and its potential. Regenerative medicine is an area of translational medical research drawing from biological theories of cell and tissue generativity, and potential for restoration of function and performance. As Kent (2012) argues, regenerative medicine builds on models of the body as regenerative including capacity for self-renewal. Self-renewal implies not just sexual reproduction, but also ‘autogeneration,’ where the role of biomedical intervention is to stimulate or control this process. Biomedical models around regenerative medicine build on broader naturalistic interpretations of bodily processes (including disease) that conceptualise the body as a natural entity that is also plastic or malleable, and therefore subjected to re-programming (Kent, 2012).

These models for interpreting bodily processes have been adopted but also re-framed by commercial businesses that use the regeneration and rejuvenation concepts to make sense of the stem cells therapies they advertise. The businesses also draw from popular discourses around translational stem cell science and construct promissory depictions of them. These depictions represent stem cells as miraculous or futuristic medical actors carrying extraordinary healing capacity. The effect is a discursive destabilisation of conventional boundaries of medicine and established medical categories, which is intertwined with representations of stem cells as having almost magical properties that collapse clear divisions between science and mythology (see also Faulkner *et al*., 2017). These destabilisations take place in two overlapping ways, which are connected with the businesses operating at the intersection of medicine and consumer culture. Firstly, the businesses are multidisciplinary and merge conventionally separate areas of medical specialisation. Secondly, they assemble conventionally distinct medical and aesthetic conditions with seemingly unrelated aetiologies under the single treatment umbrella of regenerative therapy.

Illustrative are two clinics that operate in partnership in shared facilities on London Harley Street but have separate (albeit linked) websites and separate but overlapping clinical teams: The Regenerative Clinic (2018a) and the Regenerative Beauty Clinic (2018). Both market their services under the regenerative therapy umbrella, which collates a variety of conditions and body areas that are treated. These range from musculoskeletal conditions treated at the Regenerative Clinic to aesthetic concerns treated at the Regenerative Beauty Clinic. The clinics’ primary method of therapy is trademarked Lipogems. It is marketed as ‘adipose stem cell’ therapy enabling stem cells ‘taken from your own fatty tissue, to be injected directly into sites of tissue damage, so boosting their local presence and aiding recovery.’ While the Regenerative Clinic advertises Lipogems to treat ‘problems affecting the tendons, ligaments, joints and muscles,’ the Regenerative Beauty Clinic advertises the same technique for aesthetic complaints including wrinkles, broader ‘facial rejuvenation,’ and age-related ‘restoration of facial features.’

The overlap and shared conceptual basis between the two clinics demonstrate how the idea of regeneration is used as an interpretive frame that clusters seemingly disconnected bodily conditions under a single therapeutic paradigm, providing an alternative to conventional scientific medical practice. By marketing Lipogems as a therapeutic solution to myriad musculoskeletal and aesthetic problems, the clinics shift the epistemological and diagnostic emphasis in medicine from the conventional specialisation around an object of investigation (e.g. orthopaedics, specialising in musculoskeletal conditions). They direct it, instead, towards regeneration as an epistemological and methodological framework that takes primacy over the causal origin and material location of the problem treated.

The centring of regeneration and rejuvenation and displacement of clinical areas and conditions of specialisation is further highlighted in the naming of businesses using this framework. In addition to the Regenerative- and Regenerative Beauty Clinics, UK-based businesses operate under names including the Rejuvenation Clinic (2018), Dynamic Regenerative Medicine (2015), Welsh Cell Therapy Clinic (2018), and London FAMI Clinic (2018), where FAMI (like Lipogems) is the name of the stem cell administration technique.

The collapse between seemingly unrelated medical and aesthetic complaints is also exemplified by various multi-sited, combined interventions that are marketed. The Regenerative Clinic website notes that:

Some patients who have Lipogems treatment for joint rejuvenation opt to use some of the additional fat harvested for facial rejuvenation. This treatment can be done at the same time (in one procedure) and allows patients to utilise all the harvested and treated fat.

Additionally, ‘some patients may opt to have more liposuction performed for aesthetic reason.’ This suggests that a customer may choose both liposuction through which stem cells are also harvested, combined with stem cell therapy using the stem cells simultaneously to treat musculoskeletal conditions and aesthetic complaints.

Many businesses make an extraordinary range of claims about the power of stem cells, which are said to promote effects far beyond the primary purpose for which they are administered. Vitamin Injections London (2017), for example, advertises ‘Stemcellation injections’ for anti-ageing based on sheep placenta-derived stem cells. It states that in addition to:

generating a rejuvenated complexion, Stemcellation injections have a range of health-related benefits. Stem cell injections remedy fatigue and lessen age-related health problems such as high blood pressure, diabetes, hypertension, cholesterol, gastric ulcers, migraine, blood circulation disorders and arthritis.

Such representations not only position stem cells as multi-site medico-aesthetic actors capable of healing all ills but also represent them as having almost magical properties that can bring about ‘the miracle of rejuvenation’ (Amar Clinic, 2018).

The appeal of regeneration and rejuvenation is intertwined with claims about stem cells that blur contemporary science, science fiction, and mythology. Most notable in this regard are the Genesis Clinics (2018), which market ‘stem cell facelifts’ that are claimed to:

harnesses the very life force of the universe in the form of your very own Stem Cells. Harvested, processed and concentrated into a powerful serum we call ‘Genesis Serum’ we then inject and infuse your face with up to 20 million omnipotent little builders that go to work instantly rebuilding the tissue from the inside out with new, younger cells.

Mirroring how the regeneration concept is used to cluster various therapies, the Genesis Clinics mobilise the ‘genesis’ notion to collate myriad women’s health services. These include, in addition to stem cell facelifts, aesthetic services like ‘body sculpting’ combined with conventional and unconventional sexual and reproductive health services like coil fitting alongside ‘vaginal rejuvenation’ therapies. Especially in the context of women’s health, the genesis notion associatively links the therapies with cultural ‘origin’ narratives. It taps into gendered conceptualisations of women’s bodies as the source of life because of women’s reproductive role, which is often culturally perceived to be the creation of life. By naming the clinics the Genesis Clinics and the stem cell serum they market as the genesis serum, the business constitutes women’s bodies as the source of life not only via sexual reproduction but also via autogeneration. Genesis additionally connotes mythological narratives of the origin of life by constructing an association between the therapies and biblical genesis narratives of divine creation. In relation to the genesis serum, this is the very ‘life force of the universe’ contained within (women’s) bodies in the form of stem cells.

The above highlights how science and (consumer) culture are being blurred in the marketing of stem cell therapies. The businesses forge a marketing space for stem cell therapies as a desirable biomedical lifestyle product (Saukko *et al*., 2010) by combining traditionally separated medical and aesthetic interventions as well as mythological, science fiction-like and other cultural tropes to represent stem cells’ healing power. While commercial businesses build on the science of regenerative medicine to market their products, the boundaries of what this science entails are being re-configured. This is done via future-oriented, promissory uses of science with representations of stem cells as almost omnipotent in their regenerative potential. These boundaries are also being stretched and the science of regenerative medicine commercially co-opted to accommodate (and sell to consumers) powerful cultural aspirations for medicine *as* regeneration that heals and simultaneously beautifies.

## Age Reversal

A substantial portion of the UK stem cell therapy market is focused on anti-ageing, which is aimed at achieving an aesthetically desirable youthful appearance. The anti-ageing uses draw from the regeneration and rejuvenation frameworks but also harness the intertwined idea of ‘reversal’ around ageing. Many businesses market stem cell anti-ageing therapies in ways that attribute stem cells with the ability to reverse undesirable ageing processes, represented as bodily ‘deterioration.’ These representations are linked with broader cultural discourses that frame ageing as a (societal and individual) problem for which new biotechnologies can offer a solution (Moreira, 2017). The Amar Clinic (2018) for example claims that their FAMI stem cell therapies are ‘capable of repairing the damage time has caused in the face’ by ‘restoring some of the underlying defects that are responsible for the appearance of aging’ and thus ‘restoring the facial structure to a more youthful state.’ The Pevonia medispa (2017) on the other hand proclaims that stem cells are ‘the ultimate answer to age-reversal.’

Portrayals of stem cells’ age reversing properties tap into representations of stem cells as a miraculous creative force, but additionally depict them as the fountain of youth (see also Rachul *et al*., 2015). Many businesses make reference to turning back time or repairing what is represented as damage caused by time, which represents time as a linear degenerative process moving progressively from a youthful state of health and beauty towards a damaged state of old age. This representation is significantly gendered: most anti-ageing stem cell therapies are targeted at women and foregrounded by the broader socio-cultural naturalisation of youthful femininity as the norm of female embodiment. The gendered normativity of youth and beauty, in turn, depicts the signs of ageing as damage to the natural state of womanhood and femininity, which is presumed to be young. This gendered dynamic has been well-analysed by others, especially in relation to the medicalisation of menopause as deterioration requiring treatment (Bell, 1987; Meyer, 2001; Roberts, 2007).

These discourses around stem cell therapies translate to representations of ageing as progressive deterioration that can now be *reversed* with stem cells. While anti-ageing therapies are disproportionately targeted at women, several related hair restoration therapies are also targeted at men. They are marketed via similar temporal representations of decline and reversal: ‘if you are in the early stages of hair loss and you’d like to turn back time, … help is at hand. We can’t quite build a time machine for you, but we can reverse the early stages of hair loss’ (Belvedere, 2018).

Many businesses market stem cell anti-ageing therapies by reproducing wider discourses of ageing as a treatable condition in ways that muddle clear distinctions between medical and aesthetic interventions. They also build on emerging models of ‘preventative medicine’ and health ‘management’ (rather than treatment) that position health as something that can be acted on proactively. This includes by individuals themselves who can become agentic in managing their own health (e.g. Hood and Flores, 2012). Tunc Tiryaki Clinic’s (2017) advertisement for facial stem cell injections, for example, states:

Ageing … is a descent of the whole facial structure. … Facial tissues, similar to all tissues, decline, weaken and grow old during the process of ageing. … Stem cell treatment resolves the tissue loss and anatomic deformations of ageing and rejuvenates the face.

The visible signs of ageing are represented as a symptom that results from an underlying degeneration process, which can be reversed or at least managed via stem cell therapy. Indeed, the Genesis Clinics among others make reference to the importance of individuals’ ‘age management regimes’ through which degeneration is replaced with regeneration.

Stem cell age reversal is exemplary of the emergence of biomedical lifestyle products occupying a hybrid space between biomedicine and consumer culture (Saukko *et al*., 2010). While stem cell businesses build on medical(ised) models of ageing in particular, they re-frame these models around the demands of beauty culture in ways that re-configure these models by positioning stem cells as the answer to both medical and aesthetic problems of age. They do this, not only by harnessing powerful cultural tropes around the fountain of youth, but also by narrating a promise of a better, healthier and more beautiful future for those who buy into these tropes both financially and discursively.

## ‘Stem Cells are Perhaps Nature’s Best Kept Secret:’ Promissory Narratives

As other have also argued (Petersen and Seear, 2011), stem cell therapy businesses use marketing strategies that build on the future-oriented nature of discourses around translational stem cell research in particular. This is done in ways that are intertwined with appeals to stem cell therapies’ ‘naturalness.’ The promissory scientific progress narratives that businesses capitalise on are similar those that have tended to characterise biomedical innovation more generally (Brown *et al*., 2000). Businesses use marketing phrases such as ‘cutting-edge’ and ‘ground-breaking’ to describe stem cell interventions, with the Genesis Clinics (2018) proclaiming that ‘a new era of regenerative medicine is here and we are at the leading edge.’ Plastic surgeon Dr Bernard Hayot’s website (2017) not only asserts that ‘stem cell fat transfers’ are the ‘medicine of the future,’ but also represents the experimental nature of the interventions as part of their appeal. The website states that the ‘mechanisms of this tissue regeneration are still poorly understood and are the subject of many research works making these techniques of regenerative medicine even more exciting.’

Such framing stands in direct opposition to the critiques from mainstream medical authorities, who tend to position experimental stem cell therapies as exploitative and potentially dangerous due to the experimental state. By contrast, Hayot’s website represents the experimental stage as an exciting and promising period in ways that rconceptualise the therapies as tentative or in-formation (Saukko, 2018), and *therefore* appealing. In so doing, the website deploys a future-oriented rhetoric where scientific experimentation is justified by the promise of new (and better) treatments (Moreira and Palladino, 2005). Consumers can position themselves as frontrunners of science who can contribute to scientific progress by investing in and undergoing promising new, even if experimental, medical interventions. The promissory depictions of stem cell therapy promoted by commercial businesses are more generally intertwined with marketing strategies that displace possible safety and efficacy concerns around experimental therapies. They highlight, instead, the promise this experimentation carries in relation to a new era of regenerative medicine, and consumers are given the opportunity to be a part of realising this new era.

By distinguishing the newness of stem cell-based interventions from previous approaches (which are in turn represented as increasingly anachronistic), commercial businesses position translational stem cell science and derived therapies temporally into the future. This has also characterised other new biomedical technologies (see Powell *et al*., 2007). The regenerative and rejuvenating nature of stem cell therapies is distinguished from traditional surgical procedures which are represented as unnecessarily invasive and belonging, increasingly, into the past. For example, ‘orthopaedic specialist’ Charles Willis-Owen’s website (2018) contrasts the ‘amazing healing potential’ of a single stem cell injection against ‘old-fashioned techniques’ requiring multiple operations including ‘open surgery and a long and protracted recovery.’ Similarly, the Amar Clinic (2018) compares ‘the traditional face lift [which] beautifies, but it does not rejuvenate’ with stem cell injections: after undergoing such injections, ‘the patient’s face progressively rejuvenates itself, without the need of surgery.’ The clinic adds that ‘stem cells are the base of modern rejuvenation procedures, replacing facelifting.’

The temporal promissory narratives that stem cell therapy businesses put forward are also intertwined with customer testimonials. These testimonials document positive improvement after stem cell therapy, often accompanied with ‘before and after’ imagines that construct transformation stories from an unhappy before state to a happy after state. Transformation narratives are commonly used for cosmetic interventions (Jones, 2008) and aesthetic stem cell therapies are no exception. Indeed, before and after images and testimonials also occur for orthopaedic stem cell interventions, showing a diseased bone or cartilage that has purportedly been regenerated. These narratives, together with the progress and promise narratives, additionally reinforce the image that stem cell therapies enable a brighter future for those who invest in it.

Relatedly, many businesses advertise stem cell therapies by distinguishing traditional surgical interventions from purportedly natural stem cell therapies, based on the body’s own rejuvenating potential. We identified five clinics all using the phrase ‘stem cells are perhaps nature’s best kept secret.’ While stem cell therapies are represented as capable of naturally rejuvenating the body from within, ‘surgery only corrects the wrapping … but does not restore the items below’ (Amar Clinic, 2018). Representing stem cell therapies as natural alternatives to surgery also positions surgical interventions as potentially artificial (in binary opposition to natural) and the naturalness is additionally used to mitigate possible concerns over the interventions’ safety. Many businesses claim that the fact that stem cells are natural, originating within the customer’s own body, alleviates concerns over, or even ensures, their safety.

The notion of naturalness is also used in ways that muddle conventional distinctions between orthodox and alternative medicine, and medical and alternative therapies. Many businesses offer therapies (stem cell-based and otherwise) covering a wide range between well-established medical treatments and entirely un-evidenced alternative therapies, marketed based on claims about the therapies’ naturalness and future potential. The Regenerative Clinic (2018b), for example, offers PRP- and shockwave therapy (using ‘acoustic waves’ carrying ‘high energy’ through the body) in addition to stem cell therapies. All are marketed by appealing to ‘the body’s inherent capacity to heal itself.’ Together with regeneration and rejuvenation, naturalness collates stem cell-based and other therapies in ways that produces an alternative framework for understanding what therapy or treatment can amount to. I.e. harnessing natural regeneration from the body itself rather than administering interventions surgically or with conventional pharmaceutical solutions (from the outside of the body).

Yet, some clinics simultaneously sit at the borders between conventional and alternative medicine or merge them to construct hybrid models between conventional and alterative practice, including the Regenerative Clinic. It claims to ‘specialise in avoiding surgery whenever possible’ and states that regenerative therapies can be ‘an appropriate medical alternative to orthopaedic surgery.’ However, it also offers traditional surgical techniques and the website even includes an educational video about evidence-based medicine.

Future-oriented promissory narratives allow businesses to present themselves as being at the forefront of biomedicine, enabling health consumers to be part of realising a new science of regenerative healing in-formation. They also allow businesses to position themselves as alternative to conventional medical and healthcare services that is both natural and promotes scientific advancement, including via experimental but promising new therapies.

## Conclusion

DTC marketing of experimental stem cell therapies has been considered as mainly a problem of safety and efficacy due to the therapies’ experimental nature, but the proliferation of these therapies should also be analysed in the broader context in which the therapies have been commercialised and promoted as beneficial. We have therefore explored how stem cells are represented to consumers online by DTC businesses, especially the underlying epistemic frames and how they fit within the current landscape of science, medicine, and consumer culture.

Our analysis of the digitally mediated experimental stem cell therapy market in the UK shows that the boundaries around and between science and consumer culture are being re-configured. This is happening via the blurring of conventionally separate medical specialisations, distinctions between medical and cosmetics therapies and conventional and alternative medicine as well as between science, science fiction, and mythology. The epistemological and interpretative framework of regeneration and rejuvenation shifts the emphasis and causal chain of medical practice from sub-specialities and identifying aetiologies towards the single umbrella of regenerative or rejuvenation therapy. The uses of the notion of reversal in turn intertwine medical(ised) models of ageing with the demands of youth-centred commercial beauty culture.

These frameworks for conceptualising stem cell therapies are central to how their potential is represented and marketed to consumers. They are embedded within the emergence of biomedical lifestyle products that are ambiguously situated in relation to medical and consumer goods (Saukko *et al*., 2010). The emergence of these products, in turn, is part of broader digital era trends towards knowledge pluralism and (health) consumer agency enacted online, which is facilitating the proliferation of alternative interpretations and practices of medicine and health (Neff and Nafus, 2016; Saukko, 2018). It is also contributing to the re-drawing of conventional boundaries around science and medicine (Nowotny *et al*., 2001; Saukko *et al*., 2010).

Stem cell therapies are also, and relatedly, marketed via promissory narratives (Brown *et al*., 2000). These concern a new kind of natural, non-invasive regenerative medicine of the future, which is in the process of emerging via translational stem cell science to which health consumers can participate. Viewed through this lens, the experimental nature of the therapies appears to be an exciting period of possibilities and promise (rather than dangerous or suspect due to the therapies’ unproven nature). This is precisely because the therapies are tentative, and because translational regenerative medicine is still in its formative stages, which enables health consumers to be positioned as contributors to the realisation of a medical transformation. According to stem cell therapy businesses, the transformation can be achieved, among other things, by investing in experimental stem cell therapies.

The DTC stem cell therapy market primarily exists in digital spaces where businesses can freely distribute promotional content that offers alternative representations of what stem cells science can accomplish. Consumers can directly access content, interact with commercial sources, service providers, and content created by other consumers. They must, however, simultaneously navigate the often conflicting and even contradictory information provided by conventional healthcare providers and authorities promoting evidence-based medical standards. The consequence is a complex web of digitally mediated knowledge claims and discourses at the same time as market actors (from service providers to consumers) are increasingly agentic in interpreting and prioritising the value of competing epistemic models and therapy options. To gain people’s attention and trust, conventional service providers must compete with commercial businesses offering alternative options and ways to imagine the present and future of stem cell medicine.

The DTC stem cell therapy market exemplifies broader shifts in how commercial businesses and consumers are changing medical practices and re-drawing the boundaries of medicine itself via market logics and digital agency. The discursive and epistemic frames that businesses use build on and commercially harness broader cultural ideas and aspirations to construct powerful, appealing visions of what (regenerative) science could be(come). They promise something new and better to for health consumer to invest in.

It is consequently not sufficient to presume the value of evidence-based biomedicine in delineating what the problem around experimental stem cell therapies actually is. Indeed, how we delineate and frame the problem shapes the kinds of responses that are developed to address it. We must also look beyond issues of safety, efficacy, etc. towards the digitally mediated discursive and epistemic contexts in which these therapies have proliferated to understand and appropriately respond to them. As Marres (2018, p. 441) has argued, in ‘today’s dynamic and diverse public spheres, epistemic authority will … have to be earned the hard way, through an exchange between epistemically diverse viewpoints.’ It is only by accounting for this diversity that we can begin to assess how we can or should respond to alternative commercial healthcare including experimental stem cell therapies.

## Notes

1 ‘Healthcare provider’ here broadly means public, private, licensed, and un-licensed individuals and organisation providing healthcare.

2 Notable exception is Rachul *et al*. (2015).

3 We identified no pay-to-participate clinical trials.

4 This was the case for six clinics: Regenerative Clinic, Regenerative Beauty Clinic, Welsh Cell Therapy Clinic, London FAMI Clinic, Kerastem, and Dynamic Regenerative Medicine.
